# An analytical study of active earth pressure in cohesive soil considering interlayer shear stress

**DOI:** 10.1371/journal.pone.0317293

**Published:** 2025-01-13

**Authors:** Qizhuo Liu, Tianci Zhong, Daocai Li, Yuanyuan Duan

**Affiliations:** Ltd Project Construction Management Company, Jiangxi Provincial Communications Investment Group Co., Nanchang, China; Universiti Teknologi Petronas: Universiti Teknologi PETRONAS, MALAYSIA

## Abstract

The impact of interlayer shear stress on the distribution of earth pressure in cohesive soil is notable, but currently, there lacks a comprehensive theory that integrates this factor in the calculation of active earth pressure. Drawing from the Mohr stress circle specific to clay soils, a formula to calculate interlayer shear stress has been derived. Moreover, a robust model has been formulated to compute the active earth pressure in clay soils, incorporating elements such as interlayer shear stress, effects of displacement, soil arching, and the morphology of the sliding surface. To address the challenge of integrating interlayer shear stress in clay soils for an explicit solution, a numerical iteration framework was developed. This framework facilitates the calculation of the strength, resultant force, and point of action for the active earth pressure in cohesive soil. The efficacy of this solution was evaluated by comparing it with the Rankine solution, other existing analytical solutions, and outcomes from standard model tests. Notably, when compared with the experimental findings of the word previous study, this new method exhibited a higher congruence, with discrepancies no greater than 9.8%. This indicates a significant enhancement in accuracy, providing a methodological advancement in calculating earth pressure from static to ultimate active states, inclusive of non-limit active earth pressure during controlled wall displacement scenarios. This novel approach not only supplements but also refines the theoretical framework for earth pressure calculations, offering a more precise computational tool for practical engineering applications.

## 1. Introduction

Rigid retaining walls are commonly utilized, and accurately calculating the earth pressure exerted on these walls is essential for structural design. Typically, classical earth pressure theories are employed, assuming that wall displacement reaches a limit state. However, in practical engineering scenarios, displacement of walls is often artificially restricted, and in some cases, the displacement does not reach the limit state, leading to non-limit earth pressures impacting the walls [1–4]. Utilizing classical earth pressure theories under these conditions may result in an underestimation of active earth pressure and an overestimation of the safety factors for retaining structures. It is therefore crucial to develop a methodology that accounts for the actual wall displacement when calculating active earth pressure.

The methodologies for calculating non-limit active earth pressure are categorized into two main types: The first relies on active earth pressure distribution curves derived from model tests and employs functional fitting techniques to ascertain the relationship between active earth pressure and wall displacement [5]. The second approach suggests that soil strength parameters progressively influence as the wall displaces, establishing a functional correlation between soil strength parameters and wall displacement to define the relationship between active earth pressure and wall displacement [6]. This latter method, characterized by a robust physical mechanics model and broadly applicable outcomes, has become the preferred approach for estimating non-limit earth pressures. Translation Mode (T-mode) as a typical displacement pattern for rigid retaining walls has undergone extensive research regarding its active earth pressure calculation method. For non-cohesive soils, Lu et al. [7], drawing on triaxial test results from unloading stress paths, formulated a preliminary method for calculating non-limit active earth pressure; Zhou et al. [8] addressed principal stress deviation under soil arching effects; Liu et al. [9, 10] evaluated the influence of interlayer shear stress on earth pressure distribution; Chen et al. [11] integrated prior research to develop a method for calculating non-limit active earth pressure in non-cohesive soils considering multiple factors, which aligns closely with model test results. For cohesive soils, Tu et al. [12] and Xu et al. [13], using the coordinate translation method, developed techniques for estimating active earth pressure under soil arching and displacement influences; Lou [14] expanded on this to Formulate a method for non-limit active earth pressure in cohesive soils under soil arching effects; Chen et al. [15], utilizing the variational method, created a technique for calculating active earth pressure in cohesive soils, considering tensile cracks under a logarithmic spiral slip surface. These advancements contribute to the ongoing evolution of non-limit active earth pressure calculations. In practical applications, the wall back is often uneven, creating friction between the wall and the soil. According to the principle of mutual shear stress, interlayer shear stress also exists. Studies on non-cohesive soils reveal that neglecting interlayer shear stress can lead to inaccuracies in the calculated strength distribution of active earth pressure and shifts in the point of action of the resultant force [16]. However, strategies to compute active earth pressure in cohesive soils that include interlayer shear stress remain undeveloped.

This paper enhances these methodologies by incorporating interlayer shear stress into the mechanical models for cohesive soils, establishing a numerical iterative framework that accurately calculates the force distributions, the resultant forces, and their points of action. The validation of this model through comparisons with model tests, existing analytical solutions, and traditional Rankine solutions confirms the enhanced accuracy and applicability of the proposed method, demonstrating its superiority in the field.

## 2. Relationship between soil strength parameters and displacement

### 2.1. Relationship between internal friction angle and displacement

Xu et al. [13] assumed a hyperbolic relationship between radial stress and radial strain in the soil, deriving the functional relationship between the internal friction angle of cohesive soil and wall displacement using the coordinate translation method.

$$\sin\varphi_{m}=\frac{\left(1-R_{f}+\eta R_{f})(1+\sin\varphi )(1-K)+\eta (1+K)\sin\varphi -\eta (1-K\right)}{\left(1-R_{f}+\eta R_{f})(1+\sin\varphi )(1+K)-\eta (1+K)\sin\varphi +\eta (1-K\right)}$$ (1)

where *R*_f_ is the failure ratio, typically ranging from 0.75 to 1.0 (0.85 may be used when experimental data are unavailable) [17]; *η* represents the wall displacement ratio, which is the ratio of the wall’s horizontal displacement to the displacement required to achieve the active limit state; *K* is the coefficient of static earth pressure for cohesive soils; *φ* is the angle of internal friction of the soil at the limit state.

### 2.2. Relationship between wall-soil friction angle and displacement

Assuming that the degree of mobilization of the wall-soil friction angle *δ*_m_ is equal to the degree of mobilization of the internal friction angle *φ*_m_ the following is obtained:

$$\delta_{m}=\xi \varphi_{m}$$ (2)

where *ξ* is the proportionality coefficient between the measured wall-soil friction angle *δ* and the measured internal friction angle *φ* under the limit active state.

### 2.3. Relationship between cohesion and displacement

The relationship between cohesion *c*_m_, cohesion between wall soil *c*_wm_ and displacement is calculated by Xu et al [13].

$$\begin{array}{l} c_{m}=\frac{\tan\varphi_{m}}{\tan\varphi }c\\ c_{\mathrm{wm}}=\frac{\tan\varphi_{m}}{\tan\varphi }c_{w} \end{array}\}$$ (3)

where *c* is the cohesive force of the limit state; *c*_w_ is the cohesive force between the wall soil in the limit state.

## 3. Numerical solution for non-limit active earth pressure

### 3.1. Analysis of soil stress state considering soil arching effect

**Fig 1:**
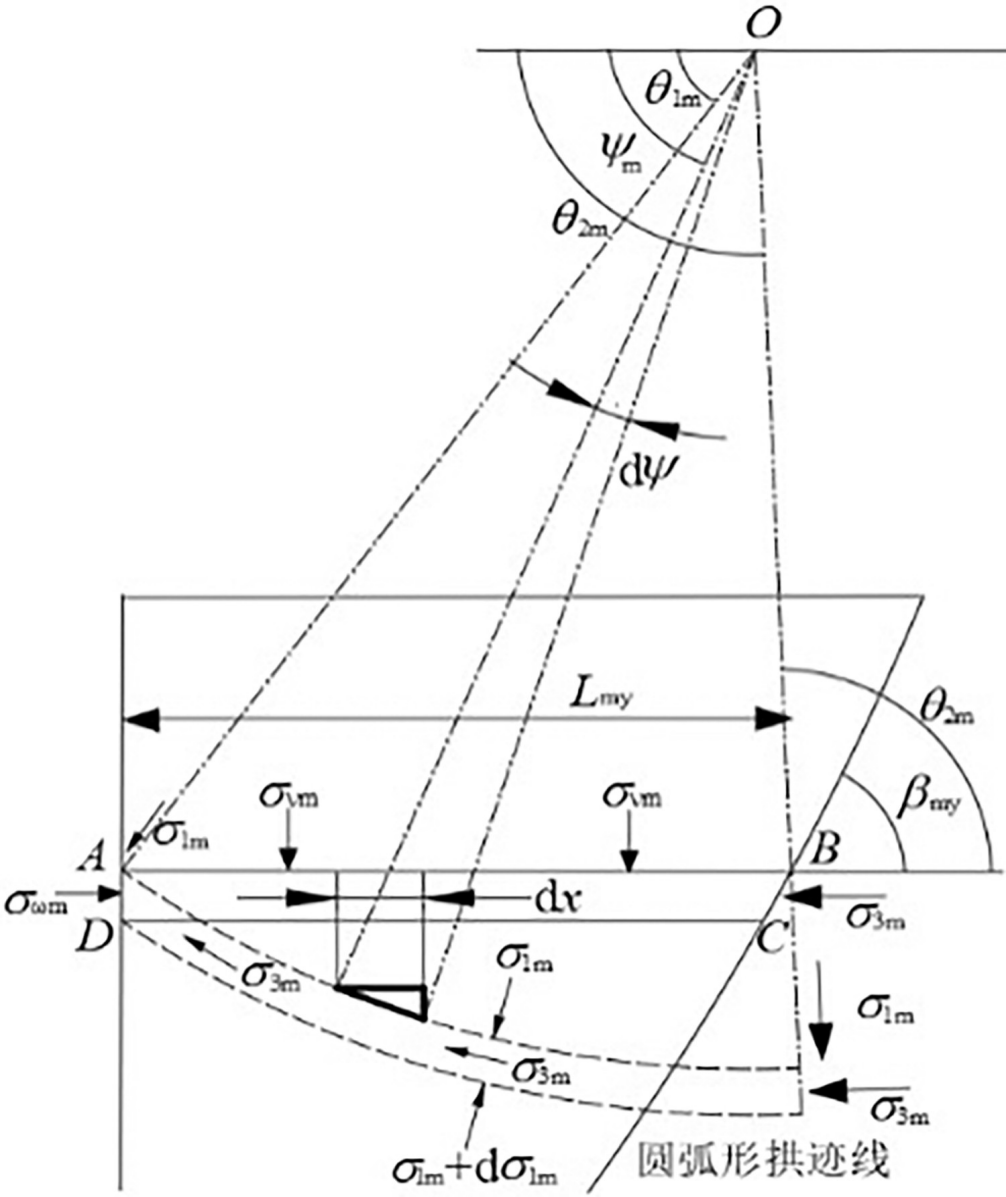
Quantitative model of soil arching effect.

The soil arching effect causes a redistribution of stress within the soil, which is the main reason for the non-linear distribution of earth pressure [18]. To quantitatively study the impact of soil arching on active earth pressure, a specific arch trace line shape is typically assumed [19, 20]. In this paper, it is assumed that the minor principal stress arch trace line is a circular arc [21], as shown in Fig 1, with the center at point *O* and the polar equation as follows:

ρ(ψ)=R (4)

where *ψ* is the polar angle; R is the radius of the soil arch.

The starting point of the arch trace is on the wall-soil contact surface, and the endpoint is on the extension line of the connection between the center *O* and the sliding crack surface. The major principal stress rotation angle *θ*_1m_ at the wall surface can be determined by Eq (5–1). The major principal stress rotation angle *θ*_2m_ at the sliding crack surface is the sum of the angle between the major principal stress and the sliding crack surface and the inclination angle of the sliding crack surface, as determined by Eq (5–2).


$$\begin{array}{l} \theta_{1m}=\frac{\pi }{2}-\frac{1}{2}\arcsin\frac{\sin\delta_{m}}{\sin\varphi_{m}}+\frac{\delta_{m}}{2}\\ \theta_{2m}=\frac{\pi }{4}-\frac{\varphi_{m}}{2}+\beta_{m} \end{array}\}$$ (5)

**Fig 2:**
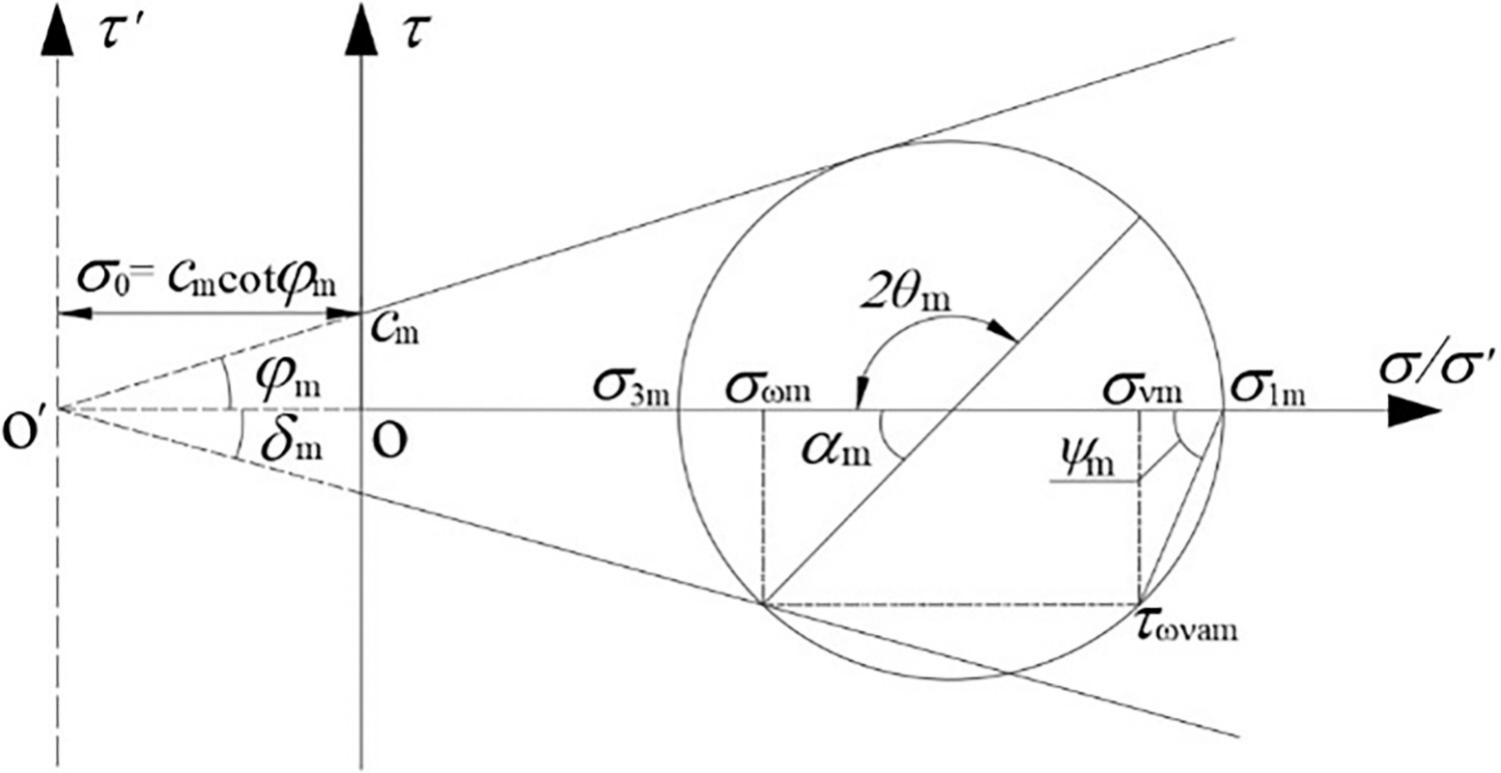
Mohr circle of stress of the soil behind retaining wall.

The stress Mohr circle of sandy soil can be obtained by translating *σ*_0_ units to the left to obtain the stress Mohr circle of clayey soil, which is the same as the nature of the soil behind the wall assumed in this paper, and the result after translation is shown in Fig 2. At this point, the horizontal stress *σ‘*_ωm_, vertical stress *σ‘*_νm_, and shear stress *τ‘*_ωνm_ at any point within the soil mass can be determined using Eq (6).

$$\begin{array}{l} \sigma {'}_{\omega m}=(\cos^{2}\theta_{1m}+K_{\mathrm{am}}\sin^{2}\theta_{1m})\sigma {'}_{1m}\\ \sigma {'}_{\mathrm{\nu m}}=(\sin^{2}\psi_{m}+K_{\mathrm{am}}\cos^{2}\psi_{m})\sigma {'}_{1m}\\ \tau {'}_{\omega \mathrm{\nu m}}=(1-K_{\mathrm{am}})\sin\psi_{m}\cos\psi_{m}\sigma {'}_{1m} \end{array}\}$$ (6)

where *ψ*_m_ is the angle between the major principal stress at any point in the soil arch and the horizontal direction, with a range of (*θ*_1m_,*θ*_2m_); *K*_am_ is the Rankine active earth pressure coefficient, determined by Eq (7).


$$K_{\mathrm{am}}=\frac{1-\sin\varphi_{m}}{1+\sin\varphi_{m}}$$ (7)

### 3.2. Calculation of lateral earth pressure coefficient

The soil arching effect causes the principal stresses within the soil to deviate, resulting in unequal vertical stresses at various points on the horizontal plane within the soil. Similar to Tu et al. [12], the vertical stresses at each point are integrated and summed, then divided by the soil layer width to obtain the average vertical stress $\bar{\sigma }{'}_{\mathrm{\nu m}}$:

$$\bar{\sigma }{'}_{\mathrm{\nu m}}=\frac{\int_{\theta_{1m}}^{\theta_{2m}}\sigma {'}_{\mathrm{\nu m}}R\sin\psi d\psi }{R(\cos\theta_{1m}-\cos\theta_{2m})}=(\frac{\left(K_{\mathrm{am}}-1)(\cos^{3}\theta_{1m}-\cos^{3}\theta_{2m}\right)}{3(\cos\theta_{1m}-\cos\theta_{2m})}+1)\sigma {'}_{1m}$$ (8)

The active side earth pressure coefficient *K*_1m_ for cohesive soil can be obtained by dividing the horizontal stress by the average vertical stress:

$$K_{1m}=\frac{\sigma {'}_{\mathrm{\omega m}}-\sigma_{0}}{\bar{\sigma }{'}_{\mathrm{\nu m}}-\sigma_{0}}=\frac{\left(\cos^{2}\theta_{m}+K_{\mathrm{am}}\sin^{2}\theta_{m})-c_{m}/((q+\gamma y)\tan\varphi_{m}+c_{m}\right)}{\frac{\left(K_{\mathrm{am}}-1)(\cos^{3}\theta_{1m}-\cos^{3}\theta_{2m}\right)}{3(\cos\theta_{1m}-\cos\theta_{2m})}+\frac{(q+\gamma y)\tan\varphi_{m}}{(q+\gamma y)\tan\varphi_{m}+c_{m}}}$$ (9)

### 3.3. Calculation of the interlayer shear stress friction coefficient

Similar to vertical stress, due to the soil arching effect, the shear stresses at various points within the soil are not equal. The shear stresses at each point are integrated and summed, then divided by the soil layer width to obtain the average shear stress $\bar{\tau }{'}_{\mathrm{\omega \nu m}}$:

$$\bar{\tau }{'}_{\mathrm{\omega \nu m}}=\frac{\int_{\theta_{1m}}^{\theta_{2m}}\tau {'}_{\mathrm{\omega \nu m}}R\sin\psi d\psi }{R(\cos\theta_{1m}-\cos\theta_{2m})}=(\frac{\left(K_{\mathrm{am}}-1)(\cos^{3}\theta_{1m}-\cos^{3}\theta_{2m}\right)}{3(\cos\theta_{1m}-\cos\theta_{2m})}+1)\sigma {'}_{1m}$$ (10)

The ratio of the *τ*_ωνm_ to ${\bar{\sigma }}_{\mathrm{\nu m}}$ is called the interlayer shear stress friction coefficient *K*_2m_:

$$K_{2m}=\frac{{\bar{\tau }}_{\mathrm{\omega \nu m}}}{\bar{\sigma }{'}_{\mathrm{\nu m}}-\sigma_{0}}=\frac{1/3(K_{\mathrm{am}}-1)(\sin^{3}\theta_{1m}-\sin^{3}\theta_{2m})/(\cos\theta_{1m}-\cos\theta_{2m})}{\frac{\left(K_{\mathrm{am}}-1)(\cos^{3}\theta_{1m}-\cos^{3}\theta_{2m}\right)}{3(\cos\theta_{1m}-\cos\theta_{2m})}+\frac{(q+\gamma y)\tan\varphi_{m}}{(q+\gamma y)\tan\varphi_{m}+c_{m}}}$$ (11)

### 3.4. Numerical iterative solution for active earth pressure

Zhu et al. [22] converted the *K*_1m_ of clayey soil into a Eq (12) using the lateral earth pressure coefficient of non-cohesive soil, addressing the issue of *K*_1m_ being difficult to integrate due to depth influence.


$$K_{1m}=\frac{\sigma {'}_{\mathrm{\omega m}}-\sigma_{0}}{{\bar{\sigma }}_{\mathrm{\nu m}}}=K_{1\mathrm{ms}}+\frac{(K_{1\mathrm{ms}}-1)\sigma_{0}}{{\bar{\sigma }}_{\mathrm{\nu m}}}$$ (12)

This paper converts the shear stress coefficient *K*_2m_ from Eq (12) to Eq (13). Since the shear stress *τ*_ωνm_ involves differentiation, its differential form is given by Eq (14). This approach eliminates *K*_2m_ but introduces a new differential form *dσ*_0_. The ratio of *dσ*_0_ to the vertical stress *σ*_vm_ still depends on the depth *y*, making it impossible to obtain an explicit solution. Therefore, when considering interlayer shear stress, the active earth pressure must be determined using numerical iteration methods.


$$K_{2m}=\frac{{\bar{\tau }}_{\mathrm{\omega \nu m}}}{{\bar{\sigma }}_{\mathrm{\nu m}}}=K_{2\mathrm{ms}}+\frac{K_{2\mathrm{ms}}\sigma_{0}}{{\bar{\sigma }}_{\mathrm{\nu m}}}$$ (13)

$$\begin{array}{l} \tau_{\mathrm{\omega \nu m}}=K_{2m}\sigma_{\mathrm{\nu m}}=K_{2\mathrm{ms}}(\sigma_{\mathrm{\nu m}}+\sigma_{0})\\ d\tau_{\mathrm{\omega \nu m}}=K_{2\mathrm{ms}}(d\sigma_{\mathrm{\nu m}}+d\sigma_{0}) \end{array}\}$$ (14)

**Fig 3:**
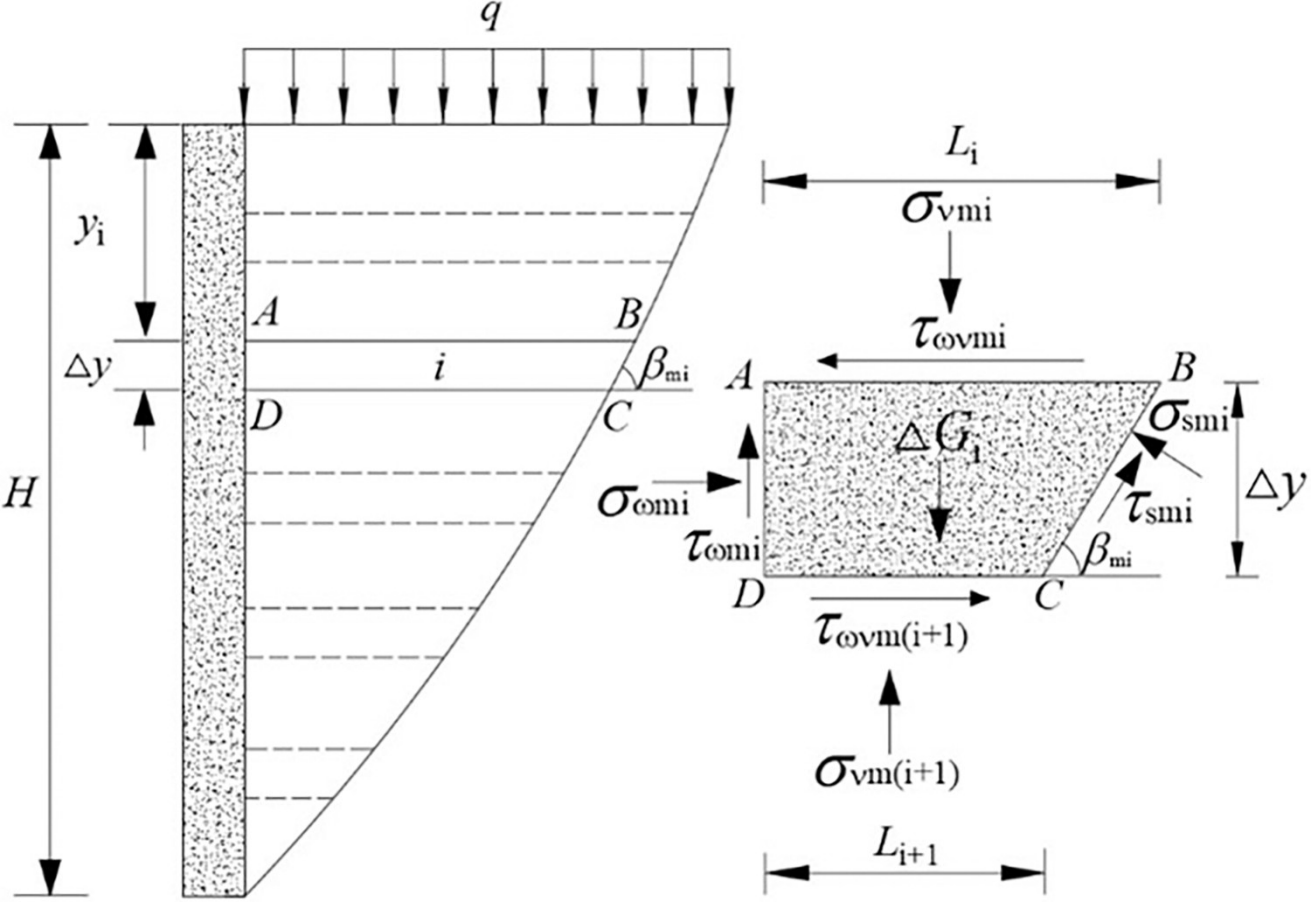
Illustration of soil mechanical model and force analysis.

This numerical iterative method divides the soil along the wall height *H* into horizontal strips with a step size △*y* as shown in Fig 3. Assuming that the calculation parameters within the same step length are the same, for example, for the *i* layer of soil, the active lateral earth pressure coefficient is *K*_1mi_ the interlayer shear stress coefficient is *K*_2mi_, and the angle of the sliding surface is *β*_mi_. Thus, using Eq (15), the normal force at the wall surface for the *i* horizontal slice *σ*_ωmi_, the tangential force at the wall surface *τ*_ωmi_, the tangential force on the sliding surface *τ*_smi_, and the interlayer shear stress *τ*_ωνmi_ can be determined.


$$\begin{array}{l} \sigma_{\mathrm{\omega mi}}=K_{1\mathrm{mi}}\sigma_{\mathrm{\nu mi}}\\ \tau_{\mathrm{\omega mi}}=\sigma_{\mathrm{\omega mi}}\tan\delta_{m}+c_{\mathrm{wm}}\\ \tau_{\mathrm{smi}}=\sigma_{\mathrm{smi}}\tan\varphi_{m}+c_{m}\\ \tau_{\mathrm{\omega \nu mi}}=K_{2\mathrm{mi}}\sigma_{\mathrm{\nu mi}} \end{array}\}$$ (15)

For the *i* horizontal slice *ABCD*, a static equilibrium analysis is performed. The sum of horizontal forces being zero gives:

$$\sigma_{\mathrm{\omega mi}}\Delta y-\tau_{\mathrm{\omega \nu m}(i+1)}L_{i+1}+\tau_{\mathrm{smi}}\cot\beta_{m}\Delta y+\tau_{\mathrm{\omega \nu mi}}L_{i}-\sigma_{\mathrm{smi}}\Delta y=0$$ (16)

The sum of vertical forces being zero results in:

$$\sigma_{\mathrm{\nu mi}}L_{i}+\Delta G_{i}-\sigma_{\mathrm{\nu m}(i+1)}L_{i+1}-\tau_{\mathrm{\omega mi}}\Delta y-\tau_{\mathrm{smi}}\Delta y-\sigma_{\mathrm{smi}}\cot\beta_{m}\Delta y=0$$ (17)

where Δ*G*_i_ is the self-weight of the *i* horizontal strip element, determined by Eq (18); *L*_i_ is the upper boundary width of the *i* horizontal strip element, determined by Eq (19); and *L*_i+1_ is the lower boundary width of the *i* horizontal strip element, calculated according to Eq (20).


$$\Delta G_{i}=\frac{\gamma (L_{i}+L_{i+1})\Delta y}{2}$$ (18)

$$L_{i}=(H-i\Delta y)\cot\beta_{\mathrm{mi}}$$ (19)

$$L_{i+1}=(H-(i+1)\Delta y)\cot\beta_{\mathrm{mi}}$$ (20)

By combining Eqs (12)–(17), an iterative format for vertical stress *σ*_νmi_ can be established:

$$\sigma_{\mathrm{\nu m}(i+1)}=\frac{\sigma_{\mathrm{\nu mi}}(L_{i}\lambda_{1\mathrm{mi}}-\Delta y\lambda_{2\mathrm{mi}})+\Delta G_{i}-\lambda_{3\mathrm{mi}}\Delta y}{L_{i+1}\lambda_{1m(i+1)}}$$ (21)

where *λ*_1mi_、*λ*_2mi_及*λ*_3mi_ are the combination coefficients during the iteration process, determined by Eq (22).


$$\begin{array}{l} \lambda_{1\mathrm{mi}}=1-K_{2\mathrm{mi}}\cot(\beta_{\mathrm{mi}}-\varphi_{m})\\ \lambda_{2\mathrm{mi}}=K_{1\mathrm{mi}}(\tan\delta_{m}+\cot(\beta_{\mathrm{mi}}-\varphi_{m}))\\ \lambda_{3\mathrm{mi}}=c_{m}(1+\cot(\beta_{\mathrm{mi}}-\varphi_{m}))\cot\beta_{\mathrm{mi}}+c_{\mathrm{wm}} \end{array}\}$$ (22)

By summing the active earth pressures of the horizontal slices from below the critical depth at the top of the wall to above the critical depth at the bottom of the wall, the resultant force of the active earth pressure can be obtained:

$$E_{\mathrm{am}}=\sum_{i=a}^{b}\sqrt{{\left(K_{1\mathrm{mi}}\sigma_{\mathrm{\nu mi}}\right)}^{2}+{\left(K_{1\mathrm{mi}}\sigma_{\mathrm{\nu mi}}\tan\delta_{m}+c_{w}\right)}^{2}}\Delta y$$ (23)

where *a* is the value of *i* at the critical depth, which can be determined using Eq (24); *b* is the value of *i* at the critical stress at the base of the wall, which is the *i* value when Eq (21) iterates to 0.


$$a=\lceil \frac{c_{m}\cot\varphi_{m}(1-\cos^{2}\theta_{m}-K_{\mathrm{am}}\sin^{2}\theta_{m})}{\gamma (\cos^{2}\theta_{m}+K_{\mathrm{am}}\sin^{2}\theta_{m})\Delta y}-\frac{q}{\gamma \Delta y}]$$ (24)

The overturning moment generated by the active earth pressure on the heel of the wall can be calculated using Eq (22).


$$M_{\mathrm{am}}=\sum_{i=a}^{b}K_{1\mathrm{mi}}\sigma_{\mathrm{\nu mi}}\Delta y(H-\frac{(2i+1)\Delta y}{2})$$ (25)

The location of the resultant force of the active earth pressure is determined by computing the ratio of the overturning moment to the resultant horizontal earth pressure and then including the distance from the critical stress point at the base of the wall to the heel.


$$h_{\mathrm{am}}=\frac{\sum_{i=a}^{b}K_{1\mathrm{mi}}\sigma_{\mathrm{\nu mi}}\Delta y(H-\frac{(2i+1)\Delta y}{2})}{\sum_{i=a}^{b}K_{1\mathrm{mi}}\sigma_{\mathrm{\nu mi}}\Delta y}+(n-b)\Delta y$$ (26)

Through the above work, a numerical iterative format for active earth pressure in clayey soil considering interlayer shear stress has been established. For practical engineering applications, based on the research findings of Tu et al. [12], Zhou et al. [23], and Yue et al. [24], the inclination angle of the Rankine sliding surface can first be determined by Eq (27), and then the strength, resultant force, overturning moment, and point of action of the active earth pressure can be solved using this iterative format. From the aforementioned derivation process, it can be seen that for curved sliding surfaces, results can also be obtained according to this solution.


$$\beta_{\mathrm{mi}}=\frac{\pi }{4}+\frac{\varphi_{m}}{2}$$ (27)

## 4. Experimental verification

**Fig 4:**
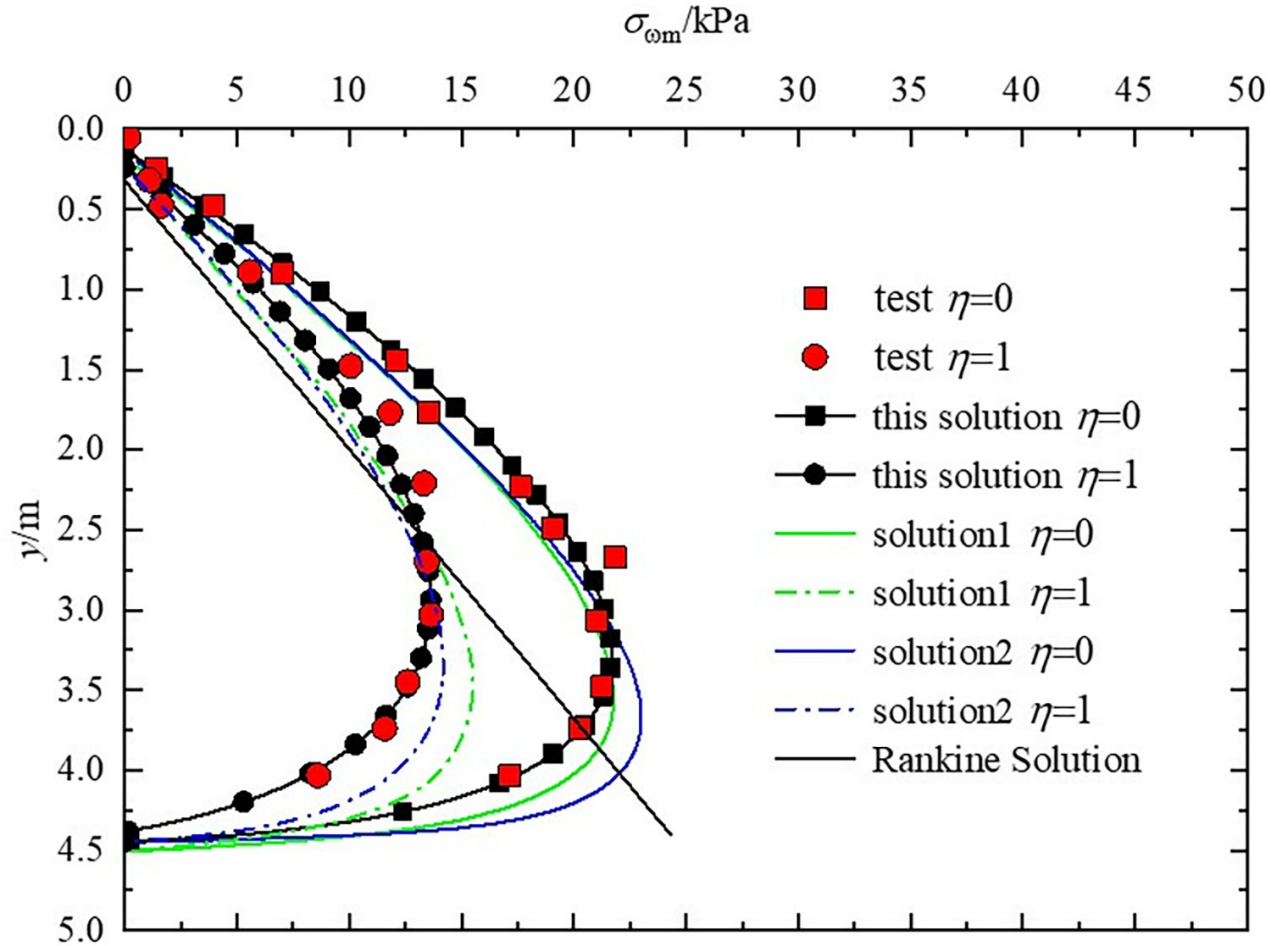
Comparison among the earth pressure distribution of this solution, model test, and two other analytic solutions.

Zhou et al. [23] conducted model tests to examine the distribution of static earth pressure (*η* = 0) and ultimate active earth pressure (*η* = 1) under the T-mode for rigid retaining walls with vertical back and horizontal backfill. The test parameters are as follows: *H* = 4.45m, *γ* = 14.27kN/m^3^, *φ* = 24.27°, *δ* = 21.4°, *c* = 1.472kPa, *c*_w_ = 0.98kPa, *q* = 0 kPa. To validate the iterative format proposed in this paper, solutions were performed using the same calculation parameters as the experiment, with a failure ratio *R*_f_ = 0.85. The active earth pressure distributions for this solution, experimental values, analytical solutions, and Rankine solutions were compared and analyzed, with the results depicted in Fig 4. Among them, Analytical Solution 1 [13] considered the effect of displacement, Analytical Solution 2 [14] further considered the soil arching effect, and this paper’s solution further considers the impact of interlayer shear stress based on these. Upon comparison, it is observed that after considering interlayer shear stress, for both static and ultimate active earth pressure, the calculations from this solution most closely match the model test results, with the maximum deviation for static earth pressure at 7.4% and for ultimate active earth pressure at 9.8%.

## 5. Conclusion

In this study, a technique is introduced for determining interlayer shear stress within clayey soils when subjected to T-mode conditions, and a computational iterative schema for non-limit active earth pressure inclusive of interlayer shear stress considerations is developed. The main findings of the study are as follows:

1. During the comparison and validation with the model tests, it was found that the numerical solution of this paper with the introduction of the interlayer shear stresses to calculate the earth pressure strength, the combined force and the point of action had a high accuracy.
2. The interlayer shear stresses will cause the earth pressure in the upper part of the wall to increase and the earth pressure in the lower part of the wall to decrease, and the overall earth pressure will show a tendency to transfer from the lower part of the wall to the upper part of the wall.
3. A maximum deviation of 7.4% for static earth pressure, which is substantially lower than that observed in methods that do not account for interlayer shear stress. This indicates a more accurate prediction by our model in static conditions.
4. For ultimate active earth pressure, where the forces are dynamic and potentially more variable, our calculated results show a deviation of up to 9.8%. Despite this being a slightly higher variance compared to static conditions, it still represents a considerable improvement in predicting the more complex scenarios of earth pressure distribution under ultimate loading conditions.

## 6. Discussion

The non-limiting soil parameters solve the problem of directly constructing the displacement-earth pressure relationship, which can basically and accurately reflect the displacement-earth pressure relationship under the corresponding state, but still need the support of simple and reliable soil ontological relationship. At the same time, the numerical solution of this paper solves the problem of difficult solution due to the change of calculation parameters with depth, and this idea can also be used to solve the non-limiting earth pressure problem of the soil strength parameters changing with depth under the rotational displacement mode, and we expect that this kind of earth pressure problem can be solved as soon as possible.

## Supporting information

S1 Data (XLSX)

## References

[1] Bang S. C. Active earth pressure behind retaining walls. Journal of Geotechnical Engineering, 1985, 111(3): 407–412.

[2] Tang Y., Chen J. G. Calculation of active earth pressure in the non-limit state based on wedge unit method. Soil Mechanics and Foundation Engineering, 2020, 56(6): 390–397.

[3] Xue K. X., Wang S. F., Hu Y. X., et al. Creep Behavior of Red-Clay Under Triaxial Compression Condition [J]. Frontiers in Earth Science, 2020, 7, Article345.

[4] Lai F., Yang D., Liu S., Zhang H., Cheng Y. Towards an improved analytical framework to estimate active earth pressure in narrow c-ϕ soils behind rotating walls about the base. Computers and Geotechnics. 2022, 141, 104544.

[5] Zhang C. G., Shan Y. P., Gao B. X. A new mathematical fitting formulation of earth pressure considering the displacement of retaining walls. Chinese Journal of Rock Mechanics and Engineering, 2021, 40(10): 2124–2135.

[6] Li M. D., Yi J. X., Zhang J. W., et al. Numerical solution for nonlimit-state earth pressure considering interlayer shear stress and the soil arching effect [J]. Computers and Geotechnics, 2023, 164, 105778.

[7] Lu K. L., Yang Y. Preliminary study of active earth pressure under nonlimit state. Rock and Soil Mechanics, 2010, 31(02): 615–619.

[8] Zhou Y. T., Chen Q. S., Chen F. Q., et al. Active earth pressure on translating rigid retaining structures considering soil arching effect. European Journal of Environmental and Civil Engineering, 2018, 22(8): 1–17.

[9] Liu Z. Y., Chen J., Li D. Y. Calculation of active earth pressure against rigid retaining wall considering shear stress. Rock and Soil Mechanics, 2016, 37(09): 2443–2450.

[10] Chen Y. B., Ke C. T., Gao H. B., et al. Non-limit state earth pressure against retaining wall considering influence of deformation. Journal of Rock Mechanics and Engineering, 2015, 34(05): 1060–1070. doi: 10.1371/journal.pone.0264690

[11] Chen J. X., Song W. W. Non-limit active earth pressure for retaining wall under translational motion. Rock and Soil Mechanics, 2019, 40(06): 2284–2292.

[12] Tu B. X., Jia J. Q. Research on active earth pressure behind rigid retaining wall from clayey backfill considering soil arching effects. Journal of Rock Mechanics and Engineering, 2012, 31(05): 1064–1070.

[13] Xu R. Q., Liao B., Wu J., et al. Computational method for active earth pressure of cohesive soil under nonlimit state. Rock and Soil Mechanics, 2013, 34(01): 148–154.

[14] Lou P. J. A method to calculate the active earth pressure with considering soil arching effect under the nonlimit state of clayey soil. Rock and Soil Mechanics, 2015, 36(04): 988–994+1014.

[15] Chen J. G., Yang Y., Chen Y. H., et al. Calculation of active earth pressure of cohesive soil behind retaining wall considering soil tensile strength. Rock and Soil Mechanics, 2020, 41(06): 1829–1835+44.

[16] Liu Z. Y., Chen J. Active earth pressure against rigid retaining wall considering shear stress under translation mode. Journal of Geotechnical Engineering, 2016, 38(12): 2254–2261.

[17] Zhao H. H. The computation of earth pressure of cohesive backfill on retaining wall. Journal of Geotechnical Engineering, 1983, (01): 134–146.

[18] Handy R. L. The arch in soil arching. Journal of Geotechnical Engineering, 1985, 111(3): 302–318.

[19] Wang J., Xia T. D., He P. F., et al. Analysis of active pressure on rigid retaining walls considering soil arching effect. Rock and Soil Mechanics, 2014, 35(07): 1914–1920.

[20] Xu R. Q., Xu Y. B., Cheng K., et al. Method to calculate active earth pressure considering soil arching effect under nonlimit state of clay. Journal of Geotechnical Engineering, 2020, 42(02): 362–371. doi: 10.11779/CJGE202002018

[21] Liu Y., Yu P. Q. Analysis of soil arch and active earth pressure on translating rigid retaining walls. Rock and Soil Mechanics, 2019, 40(02): 506–516+28.

[22] Zhu J. M., Zhao Q., Zheng Y. T. Discussion on “research on active earth pressure behind rigid retaining wall from clayey backfill considering soil arching effects”. Journal of Rock Mechanics and Engineering, 2013, 32(05): 1073–1075.

[23] Zhou Y. Y., Ren M. L. An experimental study on active earth pressure behind rigid retaining wall. Journal of Geotechnical Engineering, 1990, (02): 19–26.

[24] Yue Z. R., Peng Y. Z., Zhang S. D. Centrifuge model tests on lateral pressure on walls retaining compacted clayey backfill. Journal of Geotechnical Engineering, 1992, 14(6): 90–96.

